# Role of community health volunteers in identifying people with elevated blood pressure for diagnosis and monitoring of hypertension in Malawi: a qualitative study

**DOI:** 10.1186/s12872-021-02171-7

**Published:** 2021-07-30

**Authors:** Elvis Safary, Micrina Mwandeti, Beatrice Matanje, Claudia Beiersmann, Caroline Mtaita, Veronica Shiroya, Volker Winkler, Andreas Deckert, Pratap Kumar, Sam Phiri, Florian Neuhann

**Affiliations:** 1 Institute of Global Health, Heidelberg University, Heidelberg, Germany; 2 Lighthouse Trust, Lilongwe, Malawi; 3 Institute of Healthcare Management, Strathmore University Business School, Nairobi, Kenya; 4 Health-E-Net Limited, Nairobi, Kenya; 5 Department of Global Health, University of Washington, Seattle, USA; 6 Department of Medicine, University of North Carolina School of Medicine, Chapel Hill, NC USA; 7 School of Public Health and Family Medicine, College of Medicine, University of Malawi, Blantyre, Malawi; 8 School of Medicine and Clinical Sciences, Levy Mwanawasa Medical University, Lusaka, Zambia

**Keywords:** Community health volunteer, Hypertension, Functions, Roles, Blood pressure

## Abstract

**Background:**

In recent years, there has been greater recognition of the important role of community health volunteers in many countries and their important role informs many health programs. This include health education, provision of services such as screening, monitoring and referral to health facilities. Their roles are better understood in the areas of communicable diseases like HIV infection, Tuberculosis and Malaria however little is known about their role in non-communicable diseases. This study seeks to explore perception of CHVs’ functions, tasks, and their fulfilment in identifying people with elevated blood pressure for diagnosis and monitoring of hypertension in Lilongwe, Malawi.

**Methods:**

This was a qualitative naturalistic research design utilizing observation and semi-structured interviews with community health volunteers working in Lilongwe, Malawi. Interviews were carried out with the researcher. Participants were recruited from the ZaMaC project. An interview guide was developed with a category-guided deductive approach. The interviews were recorded through note taking. Data analysis was performed using content analysis approach.

**Results:**

Community health volunteers have multiple roles in prevention and monitoring of hypertension. They act as health educators and provide lifestyle counselling. They screened for hypertension and monitored blood pressure and assisted community members to navigate the health system such as linkage to health facilities. These roles were shaped in response to community needs.

**Conclusion:**

This study indicates the complexities of the roles of community health volunteer in identifying people with elevated BP for diagnosis and monitoring of hypertension. Understanding community health volunteers’ roles provides insight into their required competencies in provision of their daily activities as well as required training to fill in their knowledge gaps.

**Supplementary Information:**

The online version contains supplementary material available at 10.1186/s12872-021-02171-7.

## Background

Professional health care providers such as nurses and doctors are crucial for health service delivery and provision of quality care to patients. The workforce is central to advancing health and is at the heart of each and every health system [1]. However, in low-and-middle-income countries (LMIC’s) and in particular in Sub-Saharan Africa (SSA) available resources are often limited and there is inadequate workforce capacity to organize the supply and distribution of these resources [2], hence there is a need to involve community health volunteers (CHVs). Many countries in LMIC’s have established their health systems by increasing deployment of health workers including CHVs [3]. CHV are often the first level of contact for an individual, family or community within the national health system. CHVs bring health care closer to where people live and work, thus, constituting a core element of the health care process [4].

Community health volunteers (also referred to as community health workers or village health workers) have been utilized since 1980 post Alma Ata, (Alma Alta was adopted by the International Conference on Primary Health Care to protect and promote the health of all people by different stakeholders) and are formally recognized as a crucial part of the health workforce [5]. CHVs in SSA including Malawi, have provided health care to communities through a variety of roles which include community empowerment, provision of services related to HIV prevention, and linkage to health facilities [6]. For the purpose of this research paper, CHVs are defined as health workers with informal job-related training and limited in-service training who contribute to patient management at the community level. They may receive a stipend or work voluntarily in the communities in which they reside [6]. They also act as conduits for community engagement with local community leaders and health advisory committees [7].

Several non-communicable diseases interventions in sub-Saharan Africa (SSA) have been described that utilize CHV models in underprivileged communities [8, 9]. For example, studies in South Africa have highlighted the importance and varied roles of CHVs in the management of NCDs [10]. However, roles of CHVs often remain unclear and undefined as they overlap with tasks which professional health workers undertake [10]. Community health volunteer’s responsibilities have advanced over the years mainly from focusing on prevention and health promotion to more supportive roles which are associated with the increased burden of chronic conditions [11]. Engaging CHVs to screen, monitor, and refer patients can increase awareness about prevention and control of diseases in the communities.

Malawi has experienced an increase in the burden of communicable diseases such as HIV while simultaneously experiencing an increasing prevalence of non-communicable diseases (NCDs) such as hypertension and diabetes [12]. The current age-standardized prevalence of hypertension (BP estimates measured at one occasion) in Malawi was 33.2% [12]. Hypertension is more frequent among males, overweight people, and tends to increase with age [13]. Increasing NCD morbidity poses particular challenges to the healthcare sector and to the already strained health care personnel through higher numbers of patients, additional workloads, overcrowding of health facilities. and poor quality of care [14].

Given the fact that diagnosis and monitoring of hypertension requires frequent measurement and potential adjustment of treatment, current available options such as self-measurement devices or frequent visits to health facilities are not practical and feasible among disadvantaged communities. Further, this would be costly and time consuming, consequently causing additional tension to the already overloaded health facilities. Studies have suggested the use of CHVs as part of the solution to the human resource crisis in health settings [15]. Our study is part of a larger Zambia-Malawi collaboration to support local efforts in both countries to integrate NCD services into primary health care services and to provide continuous medical education (CME) to health care staff [16].

The government of the Republic of Malawi has made significant strides and shown commitment to universal health coverage (UHC) by extending its original essential health care package to include non-communicable diseases. It offers services free of charge to all Malawians within all public and private points of delivery [17]. Despite the efforts made by the government of the Republic of Malawi in managing hypertension and other NCDs, these conditions continue to be managed poorly [18] and there is a need for extended and continued care at community level through use of CHVs.

In Malawi, CHVs have mostly been utilized in programs that target infectious diseases such as tuberculosis, malaria and HIV and AIDS as well as reproductive health services. They have clearly defined roles with a body of evidence illustrating their benefits [7]. Many programs have been steered towards the aim to achieve the sustainable development goals (SDGs) 3 which includes NCDs. However, there has been little commitment to reducing NCD prevalence as evidenced by a lack of global funding [19] despite the high burden they present.

The study explored perception of CHVs’ functions, tasks, and their fulfilment in identifying people with elevated BP for diagnosis and monitoring of hypertension. The information gathered will support a meaningful response to NCDs in Malawi as well as inform stakeholders about the expansion and reduction of their roles and functions.

## Methods

### Study design

We employed a qualitative naturalistic research design, utilizing observations and semi-structured interviews to examine the daily activities of CHVs and their perception in identifying people with elevated BP for diagnosis and monitoring hypertension at community level. A naturalistic observation assumes that the roles of CHVs are socially planned and involves observing the CHVs in their natural environment whilst carrying out their health care tasks in the community as they would normally do [20, 21]. This facilitates a clear understanding of the realities CHVs are faced with while working in resource-limited settings at community level.

### Setting and study framework

The study was conducted in Lilongwe, Malawi with assistance from Lighthouse Trust (LT). The study is embedded in a collaboration between University of Heidelberg, Germany, Lighthouse Trust, Lilongwe, Malawi and CHRESO University, Lusaka Zambia. Consistent with national Universal Health Coverage (UHC) goals, the collaboration aims to support local healthcare providers in the Malawian health system to face the challenges posed by NCDs in the midst of HIV control and prolonged life spans. The project exemplarily leverages the existing HIV testing and treatment infrastructure and resources in Malawi to integrate NCD services. The overall project objective guided by the ZaMaC framework is integrating NCD prevention, screening, and treatment into routine care of HIV patients considering the context of Malawi’s health system [16].

Lilongwe district is situated in the central region of Malawi and it is estimated to have a population of 2,626,901 people based on the 2018 Malawi population and housing census. Lighthouse Trust, (World Health Organization (WHO) recognized centre of excellence for Integrated HIV prevention, treatment and care, is a registered public trust that exists to contribute to Malawi’s national response to HIV as a model in providing a continuum of high-quality care and building capacity in the health sector. LT serves a large cohort of people living with HIV and provides comprehensive routine integrated HIV prevention treatment and care [22]. Urban and peri-urban areas of greater Lilongwe were chosen because they are part of LT supported areas. LT has a network of trained CHVs who live and work in these areas. CHVs and their CBOs have already been active with Lighthouse Trust in the area of HIV prevention programs and hence this created an appropriate opportunity to introduce hypertension screening and monitoring at community level. Some of the CBO’s include Kaliyeka I, Kaliyeka II in Kawale, Ntandire and Chimoka in Area 18 and Luzi and Chisomo in Mitundu.

In our project framework, LT supports three health centres and one community hospital (Area 18, Kawale, Lumbadzi and Mitundu) in capacity building, medical and technological support, human resources, and clinical services. These facilities referred to as “prototype sites” implement project initiatives for NCD, HIV and AIDS integration as part of the collaboration. They work in close collaboration with the Malawi Ministry of Health and the CBO sites. CHVs were selected from three (Kawale, Mitundu, Area 18) of the four CBO sites supported by LH. These three CBO study sites work closely with the health facilities to facilitate and support linkage of patients between CBO sites and health facilities.

**Fig. 1:**
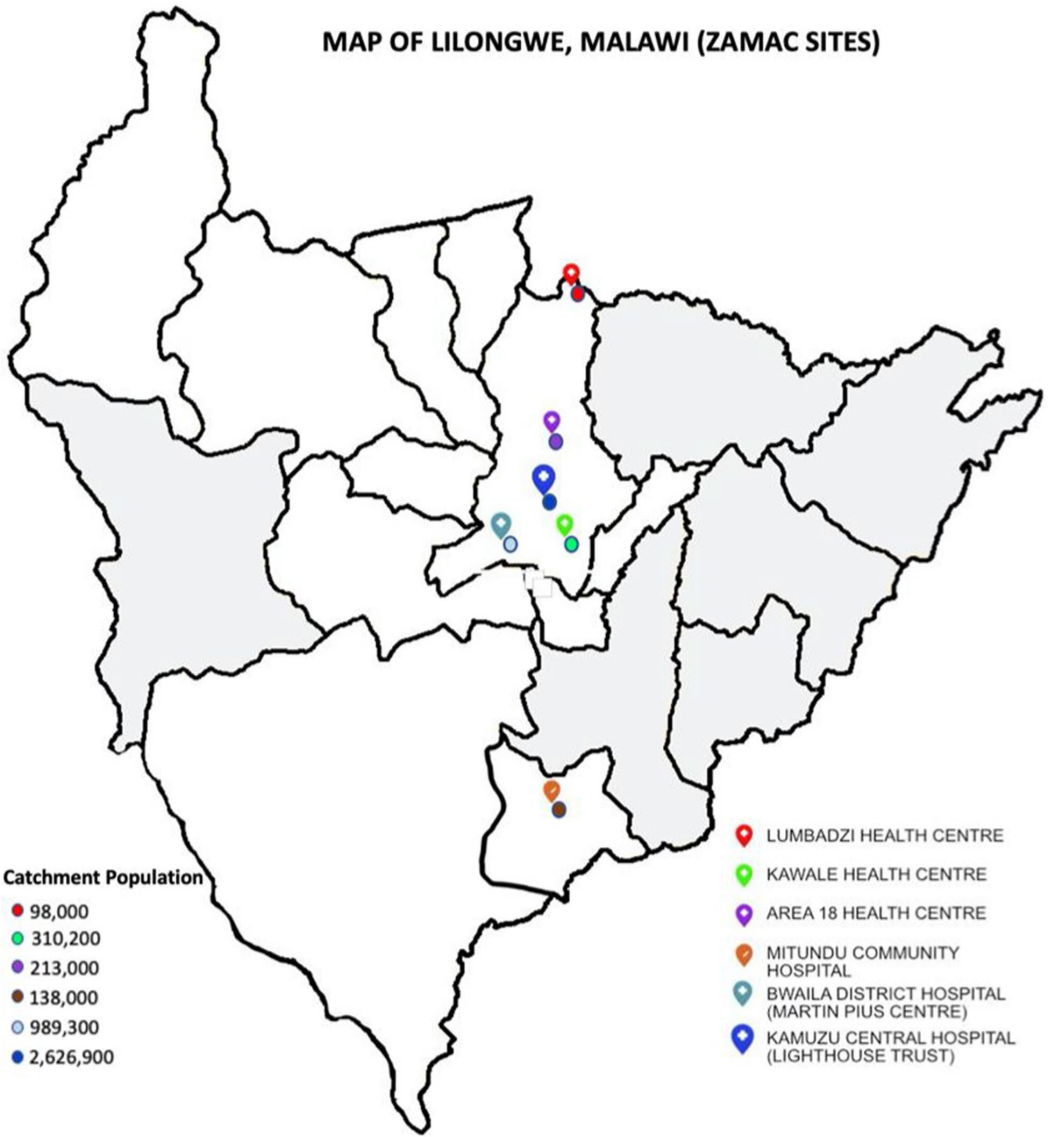
*Source*: Shiroya V. ZaMaC Health Facility Map of Lilongwe District. Heidelberg: Zambia-Malawi Collaboration (ZAMAC) Project; 2019

Figure 1 below illustrates the map of Lilongwe with all the ZaMaC sites with their respective catchment populations.

### Study participants and sampling strategy

Prior to initiation of the community service, key collaborators and project staff met to identify community needs citing NCD prevention and treatment services. The project collaborated with community-based organizations (CBOs) in Lilongwe to support the community-based model of care. The CBO sites address critical health system inputs and processes that have contributed to the implementation and expansion of community-based service delivery in Malawi. Greater Lilongwe has a high number of well-developed CBOs that enable CHVs to actively participate in community health outreach programs. Three (Kawale, Mitundu, Area 18) out of the 4 CBO sites in Lighthouse’s network were purposively selected. For each CBO site, community health volunteers were also purposively selected.

A total of 37 CHVs from three study sites (Kawale, Mitundu, Area 18) were given a one-day intensive training course on blood pressure monitoring by Lighthouse Trust. CHVs had not received any training in blood pressure monitoring before. Specifically, the training included: burden of hypertension; risk factors for hypertension, measuring blood pressure (i.e. blood pressure screenings and reading digital blood pressure machines), measuring height and weight; calculating body mass index (BMI) using BMI wheel, referring participants with elevated blood pressure of higher than 140/90 mmHg; providing lifestyle counselling, monitoring major risk factors, and reporting and follow-up procedures. Community health volunteers were then issued kits containing a bag, digital blood pressure machine, rubber stamp, weight scale, height board, and reporting books.

### Pre-defined tasks of community volunteers

Community health volunteers were required to perform the following tasks at their respective CBOs as outlined in Table 1 below. Emphasis was put on standardized documentation using a rubber stamp template [23].

**Table 1:** Tasks of CHVs and their descriptsions

Tasks/roles	Description of the function
Lifestyle Counselling	Give health talk to community members by aid of a standardized guideline developed by LH
Screening and Monitoring	Conduct cardiovascular risk assessment and BP measurement by aid of a standard operating procedure for BP measuring
Documentation	Record findings on patient register and rubber stamp template

**Fig. 2:**
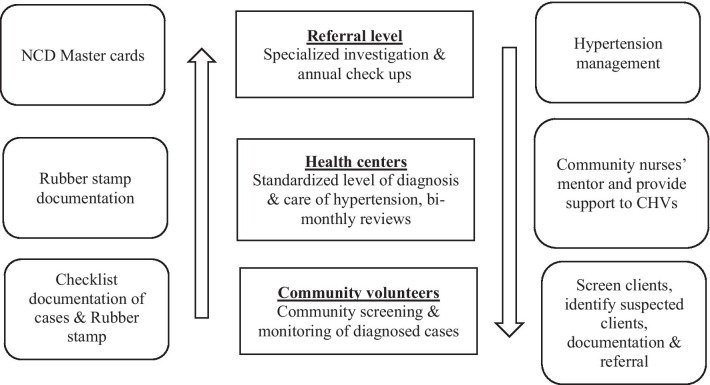
Design of the flow between the CBO/Community volunteers, health centres and tertiary level facility

The rubber stamp template is an innovative tool developed in Kenya and adopted for use in Malawi as a method of documentation. The rubber stamp template is specific to hypertension but can be tailored to other conditions. It incorporates important aspects such as blood pressure reading, risk factors, lifestyle counselling, management and follow-up. These have been customized and simplified for ease of use for community health volunteers. We used rubber stamps to print templates on paper, thus, the CHVs are able to stamp it on a paper and shade the correct findings [23]and refer clients to the health facilities as illustrated in Fig. 2.

### Interviews

The interview took place in three different community areas (Area 18, Mitundu, and Kawale) in Central Lilongwe. Each interview lasted 30–40 min and were conducted in English and sometimes in Chichewa (local language) by the community nurse in case something was not clear. Participants were asked to discuss their understanding of their pre-defined roles in screening for hypertension and monitoring with particular focus on community sensitization and prevention of hypertension, access and linkage to care and documentation.

### Observations

The main researcher and the community nurse and researcher (Chichewa speaker) conducted field observations in all the three study sites. Observations were conducted while the volunteer carried out their routine tasks. An observational study examines association of events in their natural setting without recourse to the intervention [24]. Observations targeted a set of pre-defined tasks of the CHVs including health promotion and lifestyle counselling for cardiovascular diseases (CVDs), measuring BP, calculating BMI, referring of patients identified with elevated BP based on the Standard Operating Procedures (SOPs) included as Additional file 1, supporting CHVs during NCD clinic days, and ensuring proper documentation and record keeping of data. Table 2 illustrates CHVs tasks and the observation tools employed in the study.

**Table 2:** Tasks of the CHVs and their means of verification

Tasks	Activities	Means of verification or observation tool
Lifestyle counselling	Give health talk specific on importance of intake of vegetable, fruits and low fatty diary, reduction of salt and sugar	Checked on rubber stamp template
	Explain risks	
Screening and monitoring	Take body weight and height	Check correctness on BMI chart, CVD assessment form, register & rubber stamp template
	BMI calculation using BMI chart	
	Patient positioning	
	Conduct cardiovascular risk assessment	
	BP measurement	
Management of hypertension	Lifestyle counselling	Check correctness on register/rubber stamp template
	Linkage to health facility	
	Conduct follow up	
Documentation	Record on registers and rubber stamp template	Check correctness on register/rubber stamp template

### Data collection

An assessment tool adopted from the standard operating procedures, project monitoring and evaluation framework and community document registers were used to develop an assessment form. The guiding questions focused on views about their perception of the roles pertaining to lifestyle counselling, screening and monitoring of blood pressure, referral to health facility, documentation, and fulfilment of the roles. In total, 15 community health volunteers who were present at the CBO site were interviewed and notes from 10 field observations were conducted by the main researcher and community health nurse during the three-week data collection period in June 2019 were documented through participatory observation and were interviewed through semi-structured interviews.

Observations occurred during their normal work routine. The researcher accompanied the CHVs to all activities they were conducting to be familiarized with their day-to-day work, establish rapport, and gain insight to CHVs. Additionally, based on CHVs convenience, the researcher participated in some of the activities undertaken by the CHVs such as attending routine meetings, debriefing sessions and assisting in ushering clients for screenings. The researcher collected data through note taking.

Unstructured IDIs (n = 15) were conducted with assistance from Lighthouse Trust community nurses as a way to stimulate discussions related to roles, as well as understanding the relations and linkage to health facilities. The role of the community nurse was to translate and clarify information that arose due to language barriers. These discussions were introduced during their routine work and or after observations between CHVs and their clients.

### Data analysis

Data collection, management and analysis were done concurrently. All interviews and observations were note taken. Using Microsoft word document 2017, codebooks were developed using combination of established categories based on the aim of the study guided by the ZaMaC framework of the overall project (the basic broad themes included ‘lifestyle counselling’, ‘screening and monitoring of blood pressure’, ‘referral to health facility’ and ‘documentation’) and themes that emerged from the data using a Grounded Theory approach [25]. The codebooks were flexible and themes were reviewed during data collection. To ensure a fair interpretation of the data, the transcripts were initially coded by two researchers independently. Guided by the objectives of the study, the coding process involved a critical review of each transcripts to identify the themes from the data. The two coders then met to compare their independently-identified themes. They resolved any divergence by re-reading the relevant sections of the transcripts together, and agreed on the best fit for interpretation of the data. The final step of the analysis was connecting and inter-relating themes while constructing a narration. The broad themes are discussed below, supported by relevant quotes from the transcript and notes from observational part of the study.

## Results

Principally, the CHVs had several functions and tasks, which could be summarized into four broad pre-determined roles/tasks: sensitization of screening activities, screening and monitoring clients, lifestyle counselling, referral to health facilities and documentation.

### Characteristics of community health volunteers

The group of CHVs were predominantly females, who were unpaid with several years of experience. CHV were from the communities within the three study sites. Details are summarized in Table 3.

**Table 3:** Characteristics of study participants

Characteristics	Number (%)
*Gender*	
Male	2 (13%)
Female	13 (87%)
*Age group*	
20–29	1 (7%)
30–39	4 (27%)
40–49	9 (60%)
50+	1 (7%)
*Level of education*	
Primary education (8 years)	2 (13%)
Junior certificate level (2 years)	10 (67%)
Malawi secondary certificate level (2 years)	3 (20%)
*CBO sites*	
Kawale	8 (54%)
Area 18	6 (40%)
Mitundu	1 (7%)
*Years of experience as a CHV*	
1–2 years	2 (13%)
3–4 years	9 (60%)
5+ years	4 (27%)

*CBO* Community based organization, *CHV* Community health volunteer

### Sensitisation of screening activity

Community members were informed of the BP screening activities conducted at the CBO site through various social groups as well as the community heads. None of the CBO sites had any SOPs which CHVs could follow for conducting community sensitization and outreach services.

“We do not have a formal procedure of conducting sensitization in the community, so we just inform people through our social groups, through meetings conducted by the community leader or during other activities that we conduct. We also try to inform people at the health facilities during NCD clinic days” #CHV| Female| 39 years

CHVs showed high level of readiness to take on the tasks of sensitizing the community about hypertension. The altruistic feeling was evident in their willingness to be part of sensitizing and screening the community about hypertension.

“during mother’s group meetings, we educate the women about hypertension in general, where to get blood pressure monitored at regular intervals, how to avoid it, and how to tackle it with regular consumption of medication” #CHV| Female|34 years

“even if it means extra responsibility for free [without payment], we will be satisfied by serving the community until everyone clearly understand what is hypertension. We inform our community about hypertension because we want our community to be hypertension free” #CHV| Male|36 years

### Screening and monitoring of clients

A major task for CHV was direct client service and these included screening and monitoring of clients. The first step was to identify people with elevated cardio-metabolic or cardiovascular risk.

“I visited one of the CBO on a BP screening day, on arrival, 12 clients were already seated and patiently waiting for the CHV. She introduced herself and then asked a few questions using a form to determine cardiovascular risk clients. She asked questions such as 'what is your age?’ ‘do you have history of diabetes or hypertension in your immediate family?’ ‘do you smoke or drink alcohol?” #Researcher |Fieldnotes | CBO site | day 4

It was evident that from the observation, the CHVs provided direct services to their clients which included screening for blood pressure (BP) using a digital blood pressure machine, recording it in the patient register and giving instruction for a return date for monitoring their client either weekly or monthly.

There was no consistency in identifying at risk patients because all CHVs asked different questions and even same questions were asked differently creating different meanings. In certain instances, the CHV did not even ask any question to determine cardiovascular risk patient and in other circumstances the CHV only inquired about two cardiovascular risk factors before proceeding with BP measurement. Some associated headache and stress to be a risk factor of hypertension.

“I would first inquire from patients if they have any headache or stress. Additionally, I would find out if they consume alcohol, smoke, do any exercises, if the consume food with a lot of fat and salt. These I think are the most important things to determine the risk of developing hypertension” #CHV| Male|43 years

This was also observed in the other CBO sites where CHVs were screening clients. The observation identified a gap in identification of at-risk patients for blood pressure measurement although during the training, they were all issued with a standard operating procedure for screening BP at community level. Some CHVs consistently followed SOPs for assessing cardiovascular risk among patients.

“She asked questions such as ‘what type of food do you eat/have?’ ‘how much salt do you add while cooking or after cooking?’ ‘do you exercise?’ ‘do you smoke or drink alcohol?’ ‘do you eat vegetable and fruits? How often?’ ‘do you smoke? Do you drink alcohol? If yes, can you imagine reducing alcohol consumption?” #Researcher |Fieldnotes | CBO site | day 3

Height board and weight scales were sometimes placed on surfaces that were uneven in some of the CBO sites, hence giving inconsistent results. Additionally, calculation of BMI was also omitted by all CHVs despite them having a BMI chart. These were some of the challenges observed during screening of BP.

Blood pressure was measured using an adult BP cuff size and each reading was recorded in both the recording form and client’s health passport. In all observations, patients were informed of their BP reading but no explanation was given following their readings.

### Lifestyle counselling

Almost all CHVs offered counselling to clients. The advice was given in form of a talk during which the CHVs occasionally missed important facts. Some CHVs did not have the hypertension management guideline so they gave lifestyle counselling in brief. This poses a danger of missing out on key and pertinent information that could be helpful for the community. These included but were not limited to: increasing consumption of vegetables, fruits and low-fatty dairy, advice to quit smoking, regular exercise for at least 30 min per day, reduction of weight, salt reduction to 5–6 g per day and moderate alcohol consumption. It is worth noting that some CHVs had hypertension management guideline with lifestyle counselling advice. Additionally, CHVs provided advice beyond their normal mandate on provision of food and nutrition. The following field notes recorded during a client visit at the CBO site demonstrates the CHVs role beyond the health domain.

“…after the CHV had provided lifestyle counselling to the clients, the clients went ahead to ask if the CHV provided food for the clients now that they were giving lifestyle counselling as well as nutritional advice. The CHV mentioned that the project does not offer food however she would discuss it with other CHVs in their support group and get back to them” #Researcher | Field notes | client visit | day 1

CHVs formed support groups within the community. These groups were initially formed to support people living with HIV (PLWHIV) but have been expanded to include other conditions. CHVs assumed roles as health educators in the community to provided health education within their support groups. Current education sessions within their support groups varied but included matters pertaining to hypertension and diabetes. The observation highlighted some gaps in knowledge at the same time some of them did not have standard guide for provision of health education.

However, one of the community volunteers while referring to the standard guide for provision of health education reported that more lifestyle counselling should be provided to patients particularly regarding prevention, health habits, side effect and adherence.

“we should educate them more on drinking alcohol, smoking, eating habits and exercise, I think these are the key factors affecting majority of people. people should understand the relationship between these things and hypertension. If these is done well, hypertension will be long gone” #CHV| Female|39 years

Furthermore, health education also occurred during home visits for patients with chronic illnesses who could not access CBO sites.

“sometimes they don’t come. You call them…sometimes when you call, they do not answer or their phones are off, so you are forced to go to their homes to offer counselling and find out how they are doing” #CHV| Female| 45 years

Additionally, during NCD clinic days, CHVs assisted health care staff provide to health education as well as to measure vital signs for patients before patients see the clinicians. This creates recognition and trust as they extend their duties in the communities Quote#5.

“On arrival at the health facility on an NCD day, I attended a health education session provided by a CHV. She started by introducing herself and who she was as well as what she does at the community and on what day they offer hypertension screening. The CHV went further to define what hypertension is and explained the risk factors for hypertension. Additionally, she provided lifestyle counselling as a form of Information Education and Communication (IEC) such as importance of increasing vegetable and fruit consumption, importance of physical exercise and avoidance of smoking and moderation of alcohol consumption. The information was in form of a didactic lecture with no illustration, poster or learning materials provided to patients. Questions were asked by patients and sometimes the community nurse came in to clarify on some issues” #Researcher | Field notes | NCD clinic | day 4

### Referral to health facility and CBO site

There was no clear follow-up system for referring patients to the health facility and back to the CBO site. CHVs either physically followed patients to their homes or waited for the patients to return back to the CBO site to give feedback. When patients were followed up through home visits, CHVs relied on patient’s relocation of their clinic attendance and by inspecting the health passport to confirm that they actually visited the health facility following referral. This is an informal way of implementing follow-up services, hence there is need for a follow up protocol to be put in place.

“we normally just inform them [patients] to go to the nearest health centre for further check-up and inform them to let us know what the doctor told them. Sometimes they come back to the CBO site but if they do not we have to follow them in their homes if we remember. It is not easy to follow up patients” #CHV| Female| 54 years

“ think following up patients is the most challenging task for me, sometimes you are not even sure if they went to the health facility in the first place. I normally write their names in my note book to help me remember who I referred so that I can follow up with them later” #CHV| Female| 47 years

CHVs have the responsibility to refer clients identified with elevated blood pressure to either the community nurse or the nearest health facility guided by the volunteer hypertension screening and referral standard operating procedure (included as a Additional file 1). All three health facilities (Area 18, Kawale and Mitundu) are supported by the Lighthouse Trust and have dedicated NCD days for screening, treatment, and further referral services. Referred clients had a note indicating date of visit, BP readings, lifestyle management conducted, and any other presenting complaint from the patient denoted on their health passport to present to the health provider at the health facility. Figure 2 illustrates the design of the flow between the community health volunteers, health centres and tertiary facilities.

Clients referred by CHV from the CBO sites were expected to visit health facilities for confirmation of the diagnosis of hypertension. Once confirmation had been done, patients would be put on antihypertensive drugs and treatment monitored on a monthly basis. Patients with controlled BP would then be referred back to the CBO site for monitoring which would be done on a monthly basis. However, there is no clear formal referral and follow-up procedure in place to keep track of patients who have been referred to the health facility and back to the community. Improved strategies for referral and follow-up could assist in improving the outcome of patients on treatment.

### Documentation

**Fig. 3:**
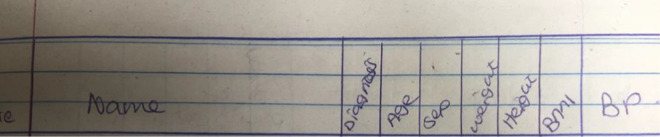
Patient register

The study revealed that documentation was often either incomplete or having missing information. CHVs documented their findings in two different documents. One register was used to register all patients who visited the CBO site and the other register was used to register patients who were identified as at-risk patients. The second register also captured other information such as patient name, age, sex, weight, height, BMI, and BP readings. Both registers are located at the CBO site as shown in Fig. 3.

Throughout the field visits, observation revealed that some of the rubber stamp templates were incorrectly filled or had unfilled gaps. When CHVs were asked, they cited lack of incompetency in shading the template. Despite the training, they did not fully understand how to complete the recording of the rubber stamp template.

“Some of us are facing difficulties in filling up the rubber stamp template. Personally, I do not fell competent enough to feel competent enough…that is why I leave some of the field blanks with the hope of getting clarification during supervision visit” #CHV | Male | 43 years

Despite this, there were also a modest realisation about one’s capacity.

“We are a grass root level health volunteers and cannot claim to be able to remember and achieve everything after one training session. We have been helping women and children in the past through other projects. We are confident that we can also serve if we get a chance to learn more about hypertension” #CHV | Female | 29 years

**Fig. 4:**
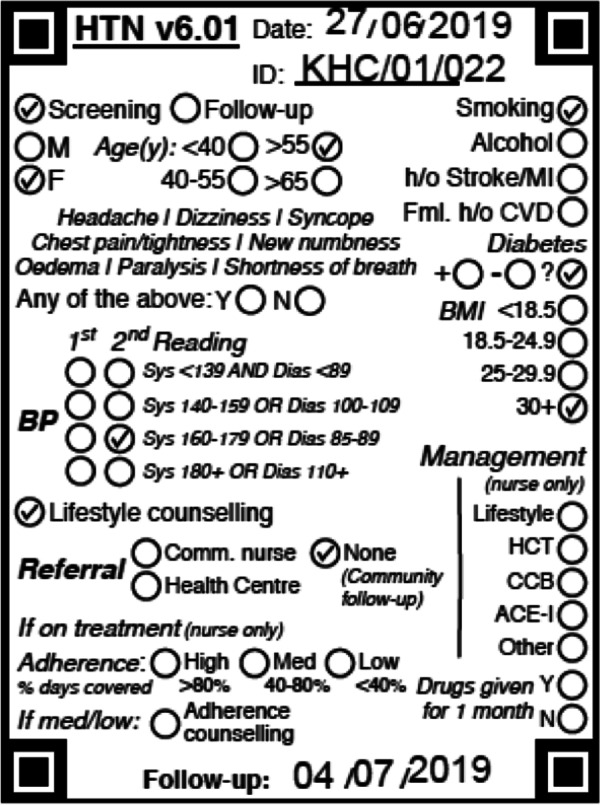
Filled rubber stamp

The rubber stamp is an innovative tool developed for improving standardised documentation at community level. The tool is a rubber stamp template that was tailor-made to capture all the information with the purposes of having a standardized document. This rubber stamp is then stamped on the patient’s health passport (Fig. 4) and filled in via shading. The rubber stamp template still requires a lot of on-going coaching and instruction on correct way to fill it in.

However, CHVs noted that the system would make patient’s management easier and save a lot of time. One CHV stated *“with the rubber stamp, it is easier to document patients’ details”* #CHV | Female | 41 years.

All information on the register were compiled on a monthly basis and copies submitted to the community nurse. The monthly reports conducted by the community nurse had a lot of incomplete or misleading information such as Body Mass Index (BMI) and Blood pressure reading.

## Discussion

The findings of this study indicate that the CHVs fulfil a variety of important roles and functions in identifying people with elevated BP for diagnosis and monitoring of hypertension at the community level. The most common tasks performed by the CHVs in our study were (1) screening community members to identify individuals with cardiovascular risk, (2) measuring blood pressure, (3) providing lifestyle counselling, and (4) referring individuals with elevated blood pressure. As such, they have a crucial role at the community level in monitoring health care in Malawi as they are very close to their communities and share the same problems and experiences as their fellow community members.

Majority of the CHVs were females and were unpaid, however, Lighthouse Trust motivates them through non-monetary means such as capacity building initiatives, exchange visits and T-shirts. Additionally, the volunteers are also formally and informally recognized for their contribution and responsibility to primary health through their position in the community, contact with health personnel and monthly provision of health education at a health facility.

Our study revealed that CHVs played an important role in identifying people with elevated BP for diagnosis and monitoring of hypertension at community level. However, some gaps were identified which can be filled to increase the success of CHVs. These inconsistencies include identification of CVD risk factors during screening, incorrect measurement of vitals (BP, weight and height), discrepancies in calculating BMI for patients, lack of clear lifestyle counselling advice, lack of clear referral and follow-up mechanisms, and incomplete or missing information in the patients registers.

The role of the CHVs in screening and monitoring patients has been seen as a crucial initial step in identifying hypertensive patients at community level. Several studies have indicated that CHVs’ role in measuring vital parameters in communicable and non-communicable diseases to be feasible and reliable [26]. Additionally, according to other studies, one of the major roles of CHVs is referral of patients to health facilities [27]. In our study, this role was clearly stipulated. CHVs referred clients identified with elevated blood pressure to either the community nurse or nearest health facility. Hypertension has been described as a symptomless but dangerous disease [28] and in such communities where health-seeking behaviour is poor and people only visit health facilities when they have serious symptoms, CHVs can play a critical role in identifying problems early enough for timely intervention.

The majority of our study findings revealed the challenges in introducing a new task for CHVs in real life without a large program backing it. Challenges can be attributed to limited literacy and limited training or formal education among CHVs, thus CHVs failed to achieve the desired diagnostic accuracy. This finding was similar to other NCDs studies conducted in other sub-Saharan Africa countries [29]. Additionally, insufficient infrastructural support and supplies as well as limited supervision and evaluation may also be factors for the gaps identified in our study. These seem to be areas which could be strengthened especially with renewed concern for universal health coverage (UHC) and the era of the sustainable development goals (SDGs).

In summary, as a priority, it is important to clearly identify and define CHVs roles and responsibilities. The observations of our study underscore the need of more structured and repeated trainings and mentorship for CHVs to enable them fulfilling their tasks e.g. with regards to appropriate lifestyle counselling and measuring waist circumference. Additionally, implementation of a community-based diagnosis and monitoring systems requires clear referral and follow-up plans agreed between communities, CHVs and health facilities. Despite the challenges observed in filling the rubber stamp, there was a high acceptance for its use among CHVs. CHVs can make good contributions to the improvement of hypertension diagnosis, identifying cardiovascular risk, and referring patients to health facilities. However, it is essential to recognize their limitations. They are not trained health workers and so cannot be expected to be as competent as professional health workers. On the other hand, their proximity to the community setting coupled with their ability to act as a bridge to existing primary health systems does offer potential to support the health system.

This study has shown great potential on how community programs can be improved and implemented through use of CHVs. Community volunteers have a broad, unique and essential role at community level. Hypertension screening, referral and follow forms only a part of the package of services offered by CHVs among others. Training, together with technical and material support, is regarded as one of the crucial factors in improving community programs [30]. There is great need to develop standardized and structured training plan for CHVs to increase their effectiveness, accuracy and ability to perform certain tasks such as correct recording and interpretation of BP findings, proper referral mechanism and use of rubber stamp and mobile phone for documentation. Regular supervisions can also be used as a strategy to address work-related challenges experienced by CHVs on a day-to-day basis thus aiding in building the capacity of CHVs.

This study does have some limitations and challenges. This was a qualitative observational study and we acknowledge that our findings may not be generalizable to the wider Malawian health care system. However, we believe that our observations can further inform the development of such interventions aimed at integrating NCDs into primary health care without the support of major programmes such as HIV or TB programmes. The work of CHVs in this study focused on people with suspected high blood pressure, thus possibly excluding patients with conditions which could have potentially benefitted from CHV services. Additionally, although the study involved three study sites, there was a relatively small sample size of CHVs from one of the CBO sites. Despite these limitations, this study is an important step in initiating the discussion on the redesigning of research to be better aligned with the communities in which the research is conducted.

## Conclusion

This study shows the complexities of the work done by CHVs working in identifying people with elevated BP for diagnosis and monitoring of hypertension at community level. Thus, defining their roles provides insight not only into the competencies required to enable them to fulfil their daily functions, but also into the type of training required to fill knowledge gaps. In communities where there is an increase in NCDs, there is a need for primary prevention and CHVs can be used in communities to identify those risks.

## Supplementary Information

**Additional file 1.** Volunteer hypertension screening and referral standard operating procedure.

## Data Availability

The full dataset (transcripts) generated and analyzed during the current study is not publicly available because they contain information that could compromise the privacy of research participants. They can be made available on request from the corresponding author due to privacy concerns of the nature of the study but can be made available from the corresponding author [ES].

## Abbreviations

BP: Blood pressure
CBO: Community Based Organization
CHV: Community Health Volunteer
HIV: Human immunodeficiency virus
NCD: Non-communicable diseases
SSA: Sub-Saharan Africa
ZaMaC: Zambia Malawi Collaboration
