# Supplementary material for: Role of community health volunteers in identifying people with elevated blood pressure for diagnosis and monitoring of hypertension in Malawi: a qualitative study
Source: BMC Cardiovasc Disord. 2021 Jul 30;21:361. doi: 10.1186/s12872-021-02171-7 (PMC8325216; doi:10.1186/s12872-021-02171-7)
Supplement: Supplementary file 1 — Additional file 1. Volunteer hypertension screening and referral standard operating procedure. [file 12872_2021_2171_MOESM1_ESM.pdf]

INTERVIEW GUIDE - Role of community health volunteers in identifying people for diagnosis and monitoring of hypertension in Malawi: a qualitative study.

Researcher: Date/Place:

To be completed by the researcher & Community health nurse & Researcher

INTRODUCTION

Good morning/afternoon/ thank you for your willingness to participate and share your experiences on regarding your role in identifying people with elevated BP for diagnosis and monitoring of hypertension conducted by community volunteers at the community level. Your thoughts and views regarding this is important in order to bring services closer to the people and improve treatment goals. Before we begin, I would like to remind you that participation in this interview is voluntary. By that I mean you are free to stop the interview at any point, you can choose not to answer a question if you are uncomfortable in doing so. All your responses will be kept confidential and will only be shared with others as part of a summary. The interview is also anonymous which means that your responses are not linked to your name or other identifying information in any way. This whole session will take approximately 45 minutes. Thank you for taking time to respond to these questions.

Before we get started, do you have any questions for me?

Questions

Experience on roles

- Can you describe the role you do within the ZaMaC project?
- How can you describe your feeling in what you do?
- How has these roles impacted on your other responsibilities?
- What changes (if any) would you make?

Experience on the community intervention

- Can you describe how you executed the following interventions?
    - Community sensitization
    - Conducting cardiovascular risks assessment
    - Lifestyle counselling
    - Referral to health facility and follow-up at community level
- What is your feeling about these interventions?
- What were the good (bad) aspects?
- What changes (if any) would you make?

Experience on the rubber stamp template

- What did you think about the rubber stamp template?
- What were the good (bad) aspects?

Closing the interview

- Is there anything else you would like to say about your experience working at the CBO?
